# Self-supervised learning for medical image classification: a systematic review and implementation guidelines

**DOI:** 10.1038/s41746-023-00811-0

**Published:** 2023-04-26

**Authors:** Shih-Cheng Huang, Anuj Pareek, Malte Jensen, Matthew P. Lungren, Serena Yeung, Akshay S. Chaudhari

**Affiliations:** 1 Department of Biomedical Data Science, Stanford University, Stanford, CA USA; 2 Center for Artificial Intelligence in Medicine & Imaging, Stanford University, Stanford, CA USA; 3 Department of Radiology, Stanford University, Stanford, CA USA; 4 Department of Computer Science, Stanford University, Stanford, CA USA; 5 Department of Electrical Engineering, Stanford University, Stanford, CA USA; 6 Clinical Excellence Research Center, Stanford University School of Medicine, Stanford, CA USA; 7 Stanford Cardiovascular Institute, Stanford University, Stanford, CA USA

**Keywords:** Medical imaging, Computer science, Scientific data

## Abstract

Advancements in deep learning and computer vision provide promising solutions for medical image analysis, potentially improving healthcare and patient outcomes. However, the prevailing paradigm of training deep learning models requires large quantities of labeled training data, which is both time-consuming and cost-prohibitive to curate for medical images. Self-supervised learning has the potential to make significant contributions to the development of robust medical imaging models through its ability to learn useful insights from copious medical datasets without labels. In this review, we provide consistent descriptions of different self-supervised learning strategies and compose a systematic review of papers published between 2012 and 2022 on PubMed, Scopus, and ArXiv that applied self-supervised learning to medical imaging classification. We screened a total of 412 relevant studies and included 79 papers for data extraction and analysis. With this comprehensive effort, we synthesize the collective knowledge of prior work and provide implementation guidelines for future researchers interested in applying self-supervised learning to their development of medical imaging classification models.

## Introduction

The utilization of medical imaging technologies has become an essential part of modern medicine, enabling diagnostic decisions and treatment planning. The importance of medical imaging is exemplified by the consistent rate of growth in medical imaging utilization in modern healthcare^1,2^. However, as the number of medical images relative to the available radiologists continues to become more disproportionate, the workload for radiologists continues to increase. Studies have shown that an average radiologist now needs to interpret one image every 3–4 s to keep up with clinical workloads^3–5^. With such an immense cognitive burden placed on radiologists, delays in diagnosis and diagnostic errors are unavoidable^6,7^. Thus, there is an urgent need to integrate automated systems into the medical imaging workflow, which will improve both efficiency and accuracy of diagnosis.

In recent years, deep learning models have demonstrated diagnostic accuracy comparable to that of human experts in narrow clinical tasks for several medical domains and imaging modalities, including chest and extremity X-rays^8–10^, computed tomography (CT)^11^, magnetic resonance imaging (MRI)^12^, whole slide images (WSI)^13,14^, and dermatology images^15^. While deep learning provides promising solutions for improving medical image interpretation, the current success has been largely dominated by supervised learning frameworks, which typically require large-scale labeled datasets to achieve high performance. However, annotating medical imaging datasets requires domain expertize, making large-scale annotations cost-prohibitive and time-consuming, which fundamentally limits building effective medical imaging models across varying clinical use cases.

Besides facing challenges with training data, most medical imaging models underperform in their ability to generalize to external institutions or when repurposed for other tasks^16^. The inability to generalize can be largely due to the process of supervised learning, which encourages the model to mainly learn features heavily correlated with specific labels rather than general features representative of the whole data distribution. This creates specialist models that can perform well only on the tasks they were trained to do^17^. In a healthcare system where a myriad of opportunities and possibilities for automation exist, it is practically impossible to curate labeled datasets for all tasks, modalities, and outcomes for training supervised models. Therefore, it is important to develop strategies for training medical artificial intelligence (AI) models that can be fine-tuned for many downstream tasks without curating large-scale labeled datasets.

Self-supervised learning (SSL), the process of training models to produce meaningful representations using unlabeled data, is a promising solution to challenges caused by difficulties in curating large-scale annotations. Unlike supervised learning, SSL can create generalist models that can be fine-tuned for many downstream tasks without large-scale labeled datasets. Self-supervised learning was first popularized in the field of natural language processing (NLP) when researchers leveraged copious amounts of unlabeled text scraped from the internet to improve the performance of their models. These pre-trained large language models^18,19^ are capable of achieving state-of-the-art results for a wide range of NLP tasks, and have shown the ability to perform well on new tasks with only a fraction of the labeled data that traditional supervised learning techniques require. Motivated by the initial success of SSL in NLP, there is great interest in translating similar techniques of SSL to computer vision tasks. Such work in computer vision has already demonstrated performance for natural images that is superior to that achieved by supervised models, especially in label-scarce scenarios^20^.

**Fig. 1:**
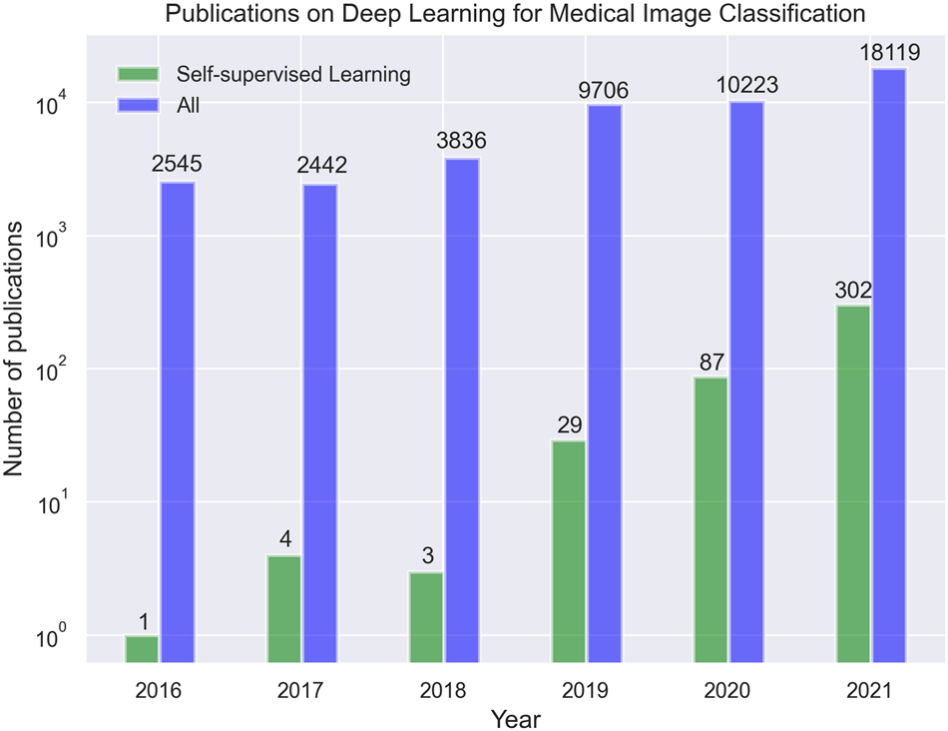
Timeline showing the number of publications on deep learning for medical image classification per year, found by using the same search criteria on PubMed, Scopus and, ArXiv. The figure shows that self-supervised learning is a rapidly growing subset of deep learning for medical imaging literature.

Reducing the number of manual annotations required to train medical imaging models will significantly reduce both the cost and time required for model development, making automated systems more accessible to different specialties and hospitals, thereby reducing workload for radiologists and potentially improving patient care. While there is already a growing trend in recent medical imaging AI literature to leverage SSL (Fig. 1), as well as a few narrative reviews^21,22^, the most suitable strategies and best practices for medical images have not been sufficiently investigated. The purpose of this work is to present a comprehensive review of deep learning models that leverage SSL for medical image classification, define and consolidate relevant terminology, and summarize the results from state-of-the-art models in relevant current literature. We focus on medical image classification tasks because many clinical tasks are based on classification, and thus our research may be directly applicable to deep learning models for clinical workflows. This review intends to help inform future modeling frameworks and serve as a reference for researchers interested in the application of SSL in medical imaging.

### Terminology and strategies in self-supervised learning

**Fig. 2:**
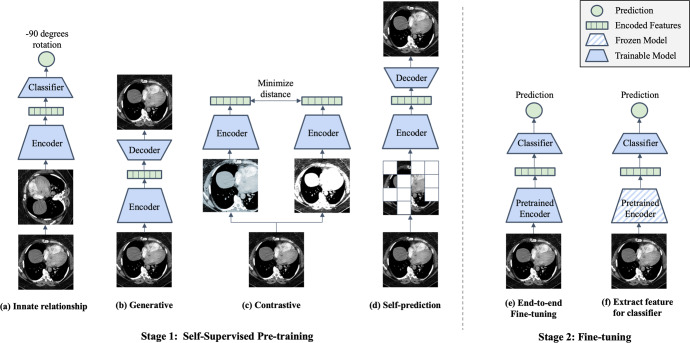
Illustration of different self-supervised learning and fine-tuning strategies. During Stage 1 a model is pre-trained using one or more of the following self-supervised learning strategies: (**a**) Innate relationship SSL pre-trains a model on a hand-crafted task by leveraging the internal structure of the data, (**b**) Generative SSL learns the distribution of training data, enabling reconstruction of the original input (**c**) Contrastive SSL forms positive pairs between different augmentations of the same image and minimizes representational distances of positive samples (**d**) Self-prediction augments or masks out random portions of an image, and reconstructs the original image based on the unaltered parts of the original image. During Stage 2, the pre-trained model can be fine-tuned using one of the following strategies: (**e**) end-to-end fine-tuning of the pre-trained model and classifier, or (**f**) train a classifier that uses extracted features from the SSL pre-trained model.

Here we provide definitions for different categorizations of self-supervision strategies, namely innate relationship, generative, contrastive, and self-prediction (Fig. 2)^23^.

**Innate relationship** SSL is the process of pre-training a model on a hand-crafted task, which can leverage the internal structure of the data, without acquiring additional labels. In the most general sense, innate relationship models perform classification or regression based on the hand-crafted task instead of optimizing based on the model’s ability to reconstruct (generative and self-prediction) or represent the original image (contrastive). Specifically, these methods are optimized using classification or regression loss derived from the given task. Pre-training the model on such a hand-crafted task makes the model learn visual features as a starting point. However, innate relationship SSL can lead to visual features that are effective only for the hand-crafted task but have limited benefits for the downstream task. Examples of innate relationship for visual inputs include predicting image rotation angle^24^, solving jigsaw puzzles of an image^25^, or determining the relative positions of image patches^26^.

**Generative** models, popularized through the advent of traditional autoencoders^27^, variational autoencoders^28^ and generative adversarial networks (GANs)^29^, are able to learn the distribution of training data, and thereby reconstruct the original input or create new synthetic data instances. By using readily available data as the target, generative models can be trained to automatically learn useful latent representations without the need for explicit labels, and they thus constitute a form of self-supervision. Early work that leverages generative models for self-supervised learning rely on autoencoders, where an encoder converts inputs into latent representations and a decoder reconstructs the representation back to the original image^30^. Subsequently, these models are optimized based on how closely the reconstructed images resemble the original image. More recent work has explored utilizing GANs for generative self-supervised learning, with improvement in performance over prior work^31,32^.

**Contrastive** self-supervised methods are based on the assumption that variations caused by transforming an image do not alter the image’s semantic meaning. Therefore, different augmentations of the same image constitute a so-called positive pair, while the other images and their augmentations are defined to be negative pairs in relation to the current instance. Subsequently a model is optimized to minimize distance in latent space between the positive pairs and push apart negative samples. Separating representations for positive and negative pairs can be based on arbitrary distance metrics incorporated into the contrastive loss function. One pioneering contrastive-based method is SimCLR^20^, which outperformed supervised models on ImageNet benchmark using 100 times fewer labels. However, SimCLR requires a very large batch size to perform well, which can be computationally prohibitive for most researchers. To reduce the large batch size required by SimCLR to ensure enough informative negative samples, Momentum Contrast (MoCo) introduced a momentum encoded queue to keep negative samples^33^. More recently, a subtype of contrastive self-supervised learning called instance discrimination, which includes methods such as DINO^34^, BYOL^35^ and SimSiam^36^, further eliminates the need for negative samples. Instead of contrastive augmented pairs from the same image, several studies have explored contrasting clustering assignments of augmented versions of the same image^37–39^.

**Self-prediction** SSL is the process of masking or augmenting portions of the input and using the unaltered portions to reconstruct the original input. The idea of self-prediction SSL originated from the field of NLP, where state-of-the-art models were pre-trained using the Masked Language Modeling approach by predicting missing words in a sentence^18,19^. Motivated by the success in NLP, early work in the field of computer vision made similar attempts by masking out or augmenting random patches of an image and training Convolutional Neural Networks (CNNs) to reconstruct the missing regions as a pre-training strategy^40^ but only with moderate success. Recently, the introduction of Vision Transformers (ViT) allowed computer vision models to also have the same transformer-based architecture. Studies such as BERT Pre-Training of Image Transformers (BEiT) and Masked Auto-encoders (MAE), which combine ViT with self-prediction pre-training objective, achieve state-of-the-art results when fine-tuned across several natural image benchmarks^41,42^. Similar to generative SSL, self-prediction models are optimized using the reconstruction loss. The key difference between self-prediction and generative SSL methods is that self-prediction applies masking or augmentations only to portions of the input image, and uses the remaining, unaltered portions to inform reconstruction. On the other hand, generative based SSL applies augmentations on the whole image and subsequently reconstruct the whole image.

There are two main strategies for fine-tuning models that have been pre-trained using SSL (Fig. 2). If we consider any imaging model to be composed of an encoder part and a classifier part, then these two strategies are (1) end-to-end fine-tuning vs. (2) extract features from the encoder first and subsequently train an additional classifier. In end-to-end fine-tuning, all the weights of the encoder and classifier are unfrozen and can be adjusted through optimization using supervised learning in the fine-tuning phase. In the feature-extraction strategy, the weights of the encoder are kept frozen to extract features as inputs to the downstream classifier. While much previous work uses linear classifiers with trainable weights (also known as linear probing), any type of classifier or architecture can be used, including Support Vector Machines (SVMs) and k-nearest neighbor^43^. It is worth emphasizing that SSL is task agnostic, and the same SSL pre-trained model can be fine-tuned for different types of downstream tasks, including classification, segmentation, and object detection.

## Results

**Fig. 3:**
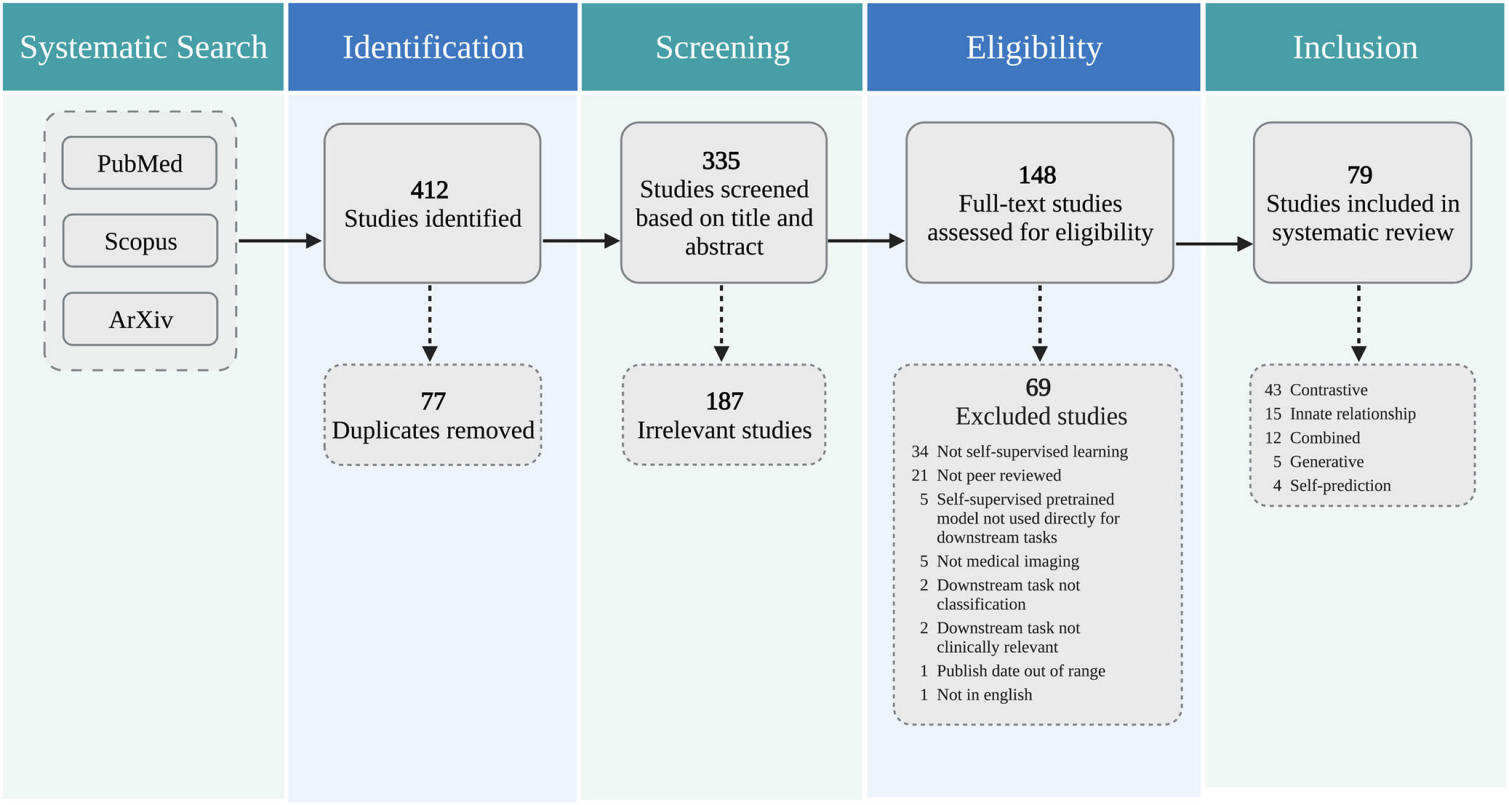
The PRISMA diagram for this review. The authors independently screened all records for eligibility. Out of 412 studies identified from PubMed, Scopus, and ArXiv, 79 studies were included in the systematic review.

**Fig. 4:**
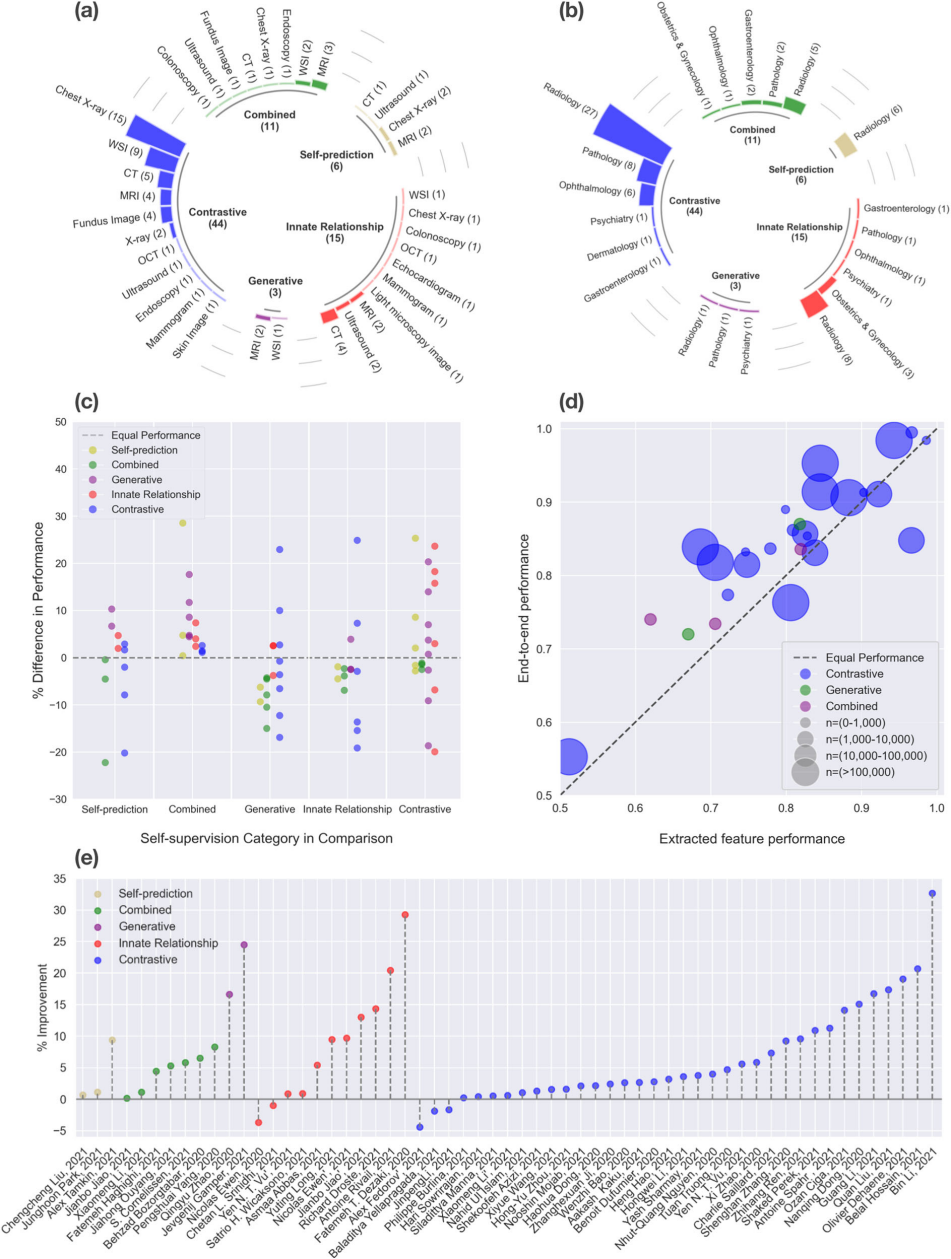
Summary of extracted data from studies in our system review. **a** Prevalence of different medical specialties split by self-supervised learning strategy. **b** Prevalence of different medical imaging modalities split by self-supervised learning strategy. **c** Relative performance difference between different types of self-supervised learning strategies on the same task. **d** Performance comparison between end-to-end fine-tuning vs. training a classifier using extracted features from pre-trained self-supervised models. **e** Relative difference in downstream task performance between self-supervised and non-self-supervised models.

A total of 412 unique studies were identified through our systematic search. After removing duplicates and excluding studies based on title and abstract using our study selection criteria (see Methods), 148 studies remained for full-text screening. A total of 79 studies fulfilled our eligibility criteria and were included for systematic review and data extraction. Figure 3 presents a flowchart of the study screening and selection process. Table 1 displays the included studies and extracted data while Fig. 4 summarizes the statistics of extracted data.

**Table 1:** Overview of studies included in our systematic review.

SSL strategy	Year	First author	Imaging modality	Clinical domain	Outcome/Task	Combined methods	SSL framework	Strategy for fine-tuning (freeze layers, end-to-end)	Metrics	SSL performance	Supervised performance	Relative difference in SSL and supervised performance
Combined	2020	Behzad Bozorgtabar^111^	Chest X-ray	Radiology	Chest abnormality	Generative, Contrastive	Autoencoder, MoCo (modified), other	Extract features from encoder -> Calculate “anomaly score” using KNN	AUROC	0.917	0.861	0.065
Combined	2020	Wan-Ting Hsieh^112^	MRI	Radiology	Cognitive Impairment and Alzheimer’s disease	Generative, Contrastive	Autoencoder, Multimodal contrastive	Extract features from encoder -> SVM	Accuracy	0.594	–	–
Combined	2020	Jianbo Jiao^53^	Ultrasound	Obstetrics & Gynecology	Standard plane detection	Contrastive, Innate Relationship	Constrastive learning, other	End-to-end	F1	0.726	0.725	0.001
Combined	2021	Yu Tian^113^	Colonoscopy	Gastroenterology	Gastrointenstinal abnormality	Contrastive, Innate Relationship	Contrastive Learning, Augmentation Prediction, Patch Position Prediction	End-to-end using unsupervised abnormality detection methods	AUROC	0.972	–	–
Combined	2021	Fatemeh Haghighi^72^	CT	Radiology	Lung nodule	Generative, Innate Relationship, Self-prediction	Autoencoder, patch pseudo label prediction, perturbed image restoration	End-to-end	AUROC	0.985	0.943	0.045
Combined	2021	Stefan Cornelissen^71^	Endoscopy	Gastroenterology	Barett’s esophagus	Self-prediction, Generative	GAN, Other	Extract features from encoder -> MLP	Accuracy	0.838	0.792	0.058
Combined	2021	Xiaomeng Li^70^	Fundus Image	Ophthalmology	Pathologic Myopia	Contrastive, Innate Relationship	Multi-view contrastive learning, rotation prediction	Extract features from encoder -> KNN	AUROC	0.991	0.98	0.011
Combined	2021	Alex Fedorov^114^	MRI	Radiology	Alzheimer’s disease	Generative, Contrastive	Autoencoder, SimCLR	Extract features from encoder -> Linear classifier	AUROC	–	–	–
Combined	2021	Jiahong Ouyang^115^	MRI	Radiology	Cognitive Impairment and Alzheimer’s disease	Contrastive, Generative	Longitudinal Neighborhood Embedding, Autoencoder	End-to-end	Accuracy	0.836	0.794	0.053
Combined	2021	Jing Ke^68^	Whole Slide Image	Pathology	Colorectal cancer, stomach cancer and breast cancer	Generative, Contrastive	CycleGAN, Contrastive Learning, Clustering	Unclear	Accuracy	0.91	–	–
Combined	2021	Pengshuai Yang^84^	Whole Slide Image	Pathology	Colorectal cancer and healthy tissue types	Generative, Contrastive	Contrastive learning, other	Extract features from encoder -> Linear classifier	Accuracy	0.914	0.844	0.083
Contrastive	2020	Hari Sowrirajan^116^	Chest X-ray	Radiology	Pleural effusion	–	MoCo	Extract features from encoder -> Linear classifier	AUROC	0.953	0.949	0.004
Contrastive	2020	Hong-Yu Zhou^117^	Chest X-ray	Radiology	Chest abnormality	–	Other	Unclear	AUROC	0.893	0.879	0.016
Contrastive	2020	Li Sun^77^	CT	Radiology	COVID-19	–	Contrastive learning	Extract features from encoder -> Linear classifier	Accuracy	0.963	0.775	0.243
Contrastive	2020	Philippe Burlina^118^	Fundus Image	Ophthalmology	Diabetic retinopathy referral	–	Deep InfoMax	Extract local features -> DeepInfoMax	AUROC	0.835	0.833	0.002
Contrastive	2020	Xiaomeng Li^119^	Fundus Image	Ophthalmology	Retinal disease	–	Multi-modal contrastive learning	Extract features from encoder -> KNN	AUROC	0.986	0.98	0.006
Contrastive	2020	Alex Fedorov^120^	MRI	Radiology	Alzheimer’s disease	–	Mutual Information Maximization	Extract features from encoder -> Linear classifier	AUROC	0.841	0.88	−0.044
Contrastive	2020	Nooshin Mojab^121^	Whole Slide Image	Ophthalmology	Glaucoma	–	SimCLR	Extract features from encoder -> Linear Classifier	Accuracy	0.923	0.904	0.021
Contrastive	2020	Bin Li^59^	Whole Slide Image	Pathology	Lung cancer and healthy tissue types	–	SimCLR	Extract features from encoder -> Multiple instance learning aggregator	AUROC	0.963	0.726	0.326
Contrastive	2020	Olivier Dehaene^88^	Whole Slide Image	Pathology	Breast cancer	–	MoCo v2	Extract features from encoder -> Multiple instance learner	AUROC	0.987	0.829	0.191
Contrastive	2020	Ozan Ciga^85^	Whole Slide Image	Pathology	Colorectal cancer and healthy tissue types	–	SimCLR	End-to-end	F1	0.914	0.801	0.141
Contrastive	2021	Colorado J Reed^122^	Chest X-ray	Radiology	Chest abnormality	–	MoCo v2	Extract features from encoder -> Linear classifier	–	–	–	–
Contrastive	2021	Fengbei Liu^123^	Chest X-ray	Radiology	Chest abnormality	–	Contrastive learning (modified)	End-to-end	AUROC	0.825	–	–
Contrastive	2021	Heng Hao^78^	Chest X-ray	Radiology	Pneumonia and COVID-19	–	SimCLR	Extract features from encoder -> Gaussian process classifer	Sensitivity	0.936	0.907	0.032
Contrastive	2021	JinpengLi^79^	Chest X-ray	Radiology	COVID-19	–	SimCLR	Unclear	AUROC	0.9	0.915	−0.016
Contrastive	2021	Matej Gazda^124^	Chest X-ray	Radiology	Pneumonia	–	Contrastive Learning	Extract features from encoder -> Linear classifier	AUROC	0.977	–	–
Contrastive	2021	Nanqing Dong^80^	Chest X-ray	Radiology	COVID-19	–	MoCo	Extract features from encoder -> Linear classifier	Accuracy	0.916	0.796	0.151
Contrastive	2021	Nhut-Quang Nguyen^125^	Chest X-ray	Radiology	Pneumonia detection	–	BYOL	Unclear	AUROC	0.988	0.95	0.04
Contrastive	2021	Shekoofeh Azizi^93^	Chest X-ray	Radiology	Chest abnormality	–	SimCLR (modified)	End-to-end	AUROC	0.773	0.763	0.013
Contrastive	2021	Tuan Truong^96^	Chest X-ray	Radiology	Pneumonia	–	SimCLR, SwAV, DINO	DVME (custom) attention based model	AUROC	0.984	0.94	0.047
Contrastive	2021	Xi Zhao^126^	Chest X-ray	Radiology	Pneumonia	–	SimCLR (modified)	Extract features from encoder -> Linear classifer	AUROC	0.889	0.84	0.058
Contrastive	2021	Yen Nhi Truong Vu^103^	Chest X-ray	Radiology	Pleural effusion	–	MoCo (modified)	End-to-end	AUROC	0.906	0.858	0.056
Contrastive	2021	Zhanghexuan Ji^60^	Chest X-ray	Radiology	Chest abnormality	–	Multimodal Contrastive, Text to Region Alignment	Unclear	AUROC	0.932	0.91	0.024
Contrastive	2021	Haohua Dong^102^	CT	Radiology	Focal liver lesion	–	Contrastive learning (modified)	Extract features from encoder -> MLP	Accuracy	0.854	0.836	0.022
Contrastive	2021	Nahid Ul Islam^127^	CT	Radiology	Pulmonary embolism	–	SeLa-v2	End-to-end	AUROC	0.957	0.947	0.011
Contrastive	2021	Wenzhi Bao^128^	CT	Radiology	Gastric cancer	–	SimSiam	End-to-end	AUROC	0.975	0.95	0.026
Contrastive	2021	Guo-Zhang Jian^129^	Endoscopy	Gastroenterology	Helicobacter Pylori	–	SimCLR	Unclear	F1	0.9	−	−
Contrastive	2021	Aakash Kaku^130^	Fundus Image	Ophthalmology	Diabetic retinopathy	–	MoCo, MSE	End-to-end, and Extract features from encoder -> Lnear classifier	AUROC	0.966	0.941	0.027
Contrastive	2021	Baladitya Yellapragada^131^	Fundus Image	Ophthalmology	Macular degeneration	–	Non-Parametric Instance Discrimination	Extract features from encoder -> Weighted KNN	Accuracy	0.94	0.958	−0.019
Contrastive	2021	Shaked Perek^132^	Mammogram	Radiology	Breast cancer	–	MoCo	End-to-end	AUROC	0.754	0.68	0.109
Contrastive	2021	Benoit Dufumier^62^	MRI	Psychiatry	Schizophrenia and Bipolar	–	SimCLR (modified)	Extract features from encoder -> Linear classifier	AUROC	0.968	0.942	0.028
Contrastive	2021	Hongwei Li^133^	MRI	Radiology	Brain tumor	–	Contrastive Learning (modified)	Extract features from encoder -> SVM	Sensitivity	0.92	0.888	0.036
Contrastive	2021	Siladittya Manna^134^	MRI	Radiology	Knee injury	–	Constrastive learning (modified)	End-to-end	AUROC	0.97	0.965	0.005
Contrastive	2021	Sohini Roychowdhury^135^	OCT	Ophthalmology	Eye disease	–	SimCLR	Unclear	F1	0.999	–	–
Contrastive	2021	Zhihang Ren^136^	Skin Image	Dermatology	Skin cancer	–	BYOL	Extract features from encoder -> Linear classifer	Accuracy	0.744	0.679	0.096
Contrastive	2021	Xiyue Wang^137^	Ultrasound	Radiology	Kidney tumor	–	InfoNCE	Extract features from encoder -> Linear classifier	F1	0.829	–	–
Contrastive	2021	Charlie Saillard^138^	Whole Slide Image	Pathology	Microsatellite instability	–	MoCo	Extract features from encoder -> Multiple instance learning	AUROC	0.88	0.82	0.073
Contrastive	2021	Jiajun Li^86^	Whole Slide Image	Pathology	Colorectal cancer and healthy tissue types	–	InfoNCE	Extract features from encoder -> Linear classifier	Accuracy	0.952	–	–
Contrastive	2021	Quan Liu^139^	Whole Slide Image	Pathology	Skin cancer and healthy tissue types	–	Triplet loss (modified)	Extract features from encoder -> Linear classifier	Accuracy	0.717	0.611	0.173
Contrastive	2021	Xiyue Wang^61^	Whole Slide Image	Pathology	Breast cancer	–	BYOL	End-to-end	AUROC	0.978	0.963	0.016
Contrastive	2021	Yash Sharmay^140^	Whole Slide Image	Pathology	Celiac disease	–	SimCLR	Extract features from encoder -> Linear classifer	AUROC	0.937	0.903	0.038
Contrastive	2021	Antoine Spahr^141^	X-ray	Radiology	Musculoskeletal abnormality	–	InfoNCE	End-to-end	AUROC	0.78	0.701	0.113
Contrastive	2021	Guang Li^142^	X-ray	Radiology	Gastritis	–	Triplet Loss (modified)	Unclear	Sensitivity	0.9	0.771	0.167
Contrastive	2022	Belal Hossain^81^	Chest X-ray	Radiology	COVID-19	–	SwAV	End-to-end	Accuracy	0.992	0.957	0.037
Generative	2020	Johnathan Osin^57^	MRI	Psychiatry	Psychiatric traits	–	Autoencoder (modified)	Extract features from encoder -> Linear classifer	Accuracy	−	−	−
Generative	2020	Qingyu Zhao^58^	MRI	Radiology	Alzheimers disease	–	Autoencoder (modified)	End-to-end	Accuracy	0.87	0.746	0.166
Generative	2021	Jevgenij Gamper^56^	Whole Slide Image	Pathology	Breast cancer	–	Multiple Instance Captioning	Unclear	Accuracy	0.9	0.723	0.245
Innate Relationship	2019	Antoine Rivail^104^	OCT	Ophthalmology	AMD Progression	–	Siamese Network (modified)	Unclear	AUROC	0.784	0.651	0.204
Innate Relationship	2019	Richard Droste^54^	Ultrasound	Obstetrics & Gynecology	Standard plane detection	–	Other	End-to-end	F1	0.766	0.67	0.143
Innate Relationship	2020	Nicolas Ewen^48^	CT	Radiology	COVID-19	–	Predict horizontal flip	Train last layer, then End-to-End	AUROC	0.861	0.785	0.097
Innate Relationship	2020	Siladittya Manna^50^	MRI	Radiology	ACL Tear	–	Jigsaw Puzzle	Replace part of the pretrained model with custom architecture	AUROC	0.848	–	–
Innate Relationship	2020	Jianbo Jiao^49^	Ultrasound	Obstetrics & Gynecology	Standard plane detection	–	Reorder slices, Predict Translation	End-to-end	F1	0.757	0.67	0.13
Innate Relationship	2021	Asmaa Abbas^82^	Chest X-ray	Radiology	COVID-19	–	Autoencoder (modified)	Freeze low-level layers, fine-tune high-level layers	Accuracy	0.975	0.925	0.054
Innate Relationship	2021	Anuja Vats^44^	Colonoscopy	Gastroenterology	GI abnormality	–	Rotation Prediction	End-to-end	Accuracy	0.6	–	–
Innate Relationship	2021	Nicolas Ewen^45^	CT	Radiology	COVID-19	–	Rotation Prediction	Unclear	Accuracy	0.867	0.9	-0.037
Innate Relationship	2021	Yujie Zhu^46^	CT	Radiology	COVID-19	–	Rotation Prediction, Patch Order Predicction	Unclear	–	–	–	–
Innate Relationship	2021	Yuting Long^47^	CT	Radiology	Pneumonia	–	Rotation Prediction	Extract features from encoder -> Linear Classifier	Accuracy	0.821	0.75	0.095
Innate Relationship	2021	Fatemeh Taheri Dezaki^55^	Echocardiogram	Radiology	Atrial fibrilation	–	Temporal Cycle-Consistency	Extract features from encoder -> Create similarity matrix -> CNN classifier	Accuracy	0.787	0.609	0.292
Innate Relationship	2021	Satrio Hariomurti Wicaksono^51^	Light microscopy image	Obstetrics & Gynecology	Oocyte stage	–	Jigsaw Puzzle	Unclear	Accuracy	0.919	0.911	0.009
Innate Relationship	2021	Yen Nhi Truong Vu^52^	Mammogram	Radiology	Malignancy	–	Jigsaw Puzzle	Unclear	AUROC	0.962	0.954	0.008
Innate Relationship	2021	Yuki Hashimoto^90^	MRI	Psychiatry	Schizophrenia	–	Other	Extract features from encoder -> Linear classifier	Accuracy	0.778	–	–
Innate Relationship	2021	Chetan L. Srinidhi^87^	Whole Slide Image	Pathology	Colorectal cancer and healthy tissue types	–	Other	End-to-end	F1	0.911	0.92	−0.01
Self-prediction	2021	Alex Tamkin^143^	Chest X-ray	Radiology	Chest abnormality	–	SHED	Extract features from encoder -> Linear classifier	Accuracy	0.745	0.681	0.094
Self-prediction	2021	Junghoon Park^67^	Chest X-ray	Radiology	COVID-19	–	Inpainting, Local Pixel Shuffling	End-to-end	Accuracy	0.986	0.975	0.011
Self-prediction	2021	Ananya Jana^65^	CT	Radiology	Liver fibrosis	–	Image Restoration	Extract features from encoder -> MLP	AUROC	0.847	–	–
Self-prediction	2021	Hai Zhong^64^	MRI	Radiology	Heart failure	–	Pixel shuffling, Image Restoration	End-to-end	AUROC	0.768	–	–
Self-prediction	2021	Wonsik Jung^66^	MRI	Radiology	Autism Spectrum Disorder	–	Masked Autoencoder	Unclear	AUROC	0.76	–	–
Self-prediction	2021	Chengcheng Liu^63^	Ultrasound	Radiology	Gastrointenstinal tumor	–	Image Restoration	Extract features from second last layer -> Meta attention module	AUROC	0.881	0.875	0.007

### Innate relationship

Innate relationship was used in 15 out of 79 studies (Table 1). Nine of these studies designed their innate relationship pre-text task based on different image transformations, including rotation prediction^44–47^, horizontal flip prediction^48^, reordering shuffled slices^49^, and patch order prediction^46,50–52^. Notably, Jiao et al. pre-trained their models simultaneously with two innate relationship pre-text tasks (slice order prediction and geometric transformation prediction), and showed that a weight-sharing Siamese network out-performs a single disentanged model for combining the two pre-training objectives^53^. The remaining six studies designed clinically relevant pre-text tasks by exploiting the unique properties of medical images. For instance, Droste et al. utilized a gaze tracking dataset and pre-trained a model to predict sonographers’ gazes on ultrasound video frames with gaze-point regression^54^. Dezaki et al. employed temporal and spatial consistency to produce features for echocardiograms that are strongly correlated with the heart’s inherent cyclic pattern^55^. Out of all innate relationship based studies, ten compared performance to those of supervised pre-trained models and eight of them showed improvement in performance. On average, clinically relevant pre-text tasks achieved greater improvements in performance over transformation-based pre-text tasks, when compared to purely supervised methods (13.7% vs. 5.03%).

### Generative

Generative SSL was used in 3 out of 79 studies (Table 1). Gamper et al. extracted histopathology images from textbooks and published papers along with the figure captions and devised an image captioning task for self-supervised pre-training, where a ResNet-18 was used for encoding images, and the representation was fed to transformers for image-captioning^56^. They were subsequently able to use the learned representations for a number of downstream histopathology tasks, including breast cancer classification. Osin et al.^57^ leveraged the chronology of sequential images in brain fMRI for self-supervised pre-training. Brain fMRI scans are typically acquired with subjects alternating between a passive and an active phase, where the subject is instructed to perform some task or receives some external stimulus. During the self-supervision phase, Osin et al. trained two networks: an autoencoder to generate the active fMRI image given the passive image, and an LSTM to predict the next active image. The representations learned during the self-supervision were then used to train a classifier to predict psychiatric traits such as post-traumatic stress disorder (PTSD). Finally, Zhao et al. use a generative approach with an autoencoder with an additional constraint that explicitly associates brain age to the latent representations for longitudinally acquired brain MRIs^58^. Of the three studies, two reported comparisons with purely supervised models and showed relative improvements of 16.6%^58^ and 24.5%^56^ with self-supervised learning.

### Contrastive

The majority of the studies that remained after our full-text screening (44/79) used contrastive learning as their self-supervised pre-training strategy (Table 1). SimCLR, MoCo and BYOL were the three most used frameworks, applied in 13, 8, and 3 papers, respectively. Some papers leveraged medical domain priors to create specialized strategies for creating positive pairs. For pathology slices, Li et al. exploited that the neighborhood around a patch is likely to be similar, and used pre-clustering to find dissimilar patches^59^. In radiology, Ji et al. used multimodal contrastive learning by matching X-rays with corresponding radiology reports^60^. They extracted and fused the representations of the image and text modalities through both global image-sentence matching and local attention-based region-phrase matching. Wang et al. utilized both radiomic features and deep features from the same image to form positive pairs^61^. They also utilized the spatial information of the patches, by mining positive pairs from proximate tumor areas and negative pairs from distant tumor areas. Dufumier et al. (2021) used patient meta-data from MRI to form positive pairs^62^. Thirty-six studies compared contrastive SSL pre-training to supervised pre-training and reported an average improvement in performance of 6.35%.

### Self-prediction

Self-prediction was used in six out of all included studies (Table 1). We consider studies that applied local-pixel shuffling as self-prediction since the augmentation operation, which shuffles the order of pixels, is applied only to a random patch of an image. Liu et al. used a U-net model to restore ultrasound images augmented with local-pixel shuffling, and they subsequently concatenated the outputs of the U-net encoder with featurized clinical variables (age, gender, tumor size) for the downstream prediction task^63^. Similarly, Zhong et al. designed three image restoration tasks on cine-MRI videos and used a U-net-like encoder-decoder architecture including skip connections to perform the image restoration^64^. Three different image restoration tasks were set up using local-pixel shuffling, within-frame pixel shuffling, and covering an entire video frame with random pixels. Jana et al. used an encoder-decoder architecture for image restoration of CT scans that were corrupted by swapping several small patches within a single CT slice^65^. Jung et al. created a functional connectivity matrix between pairs of region-of-interest in rs-fMRI for each subject, and created a masked auto-encoder task by randomly masking out different rows and columns of the matrix for restoration^66^. Two of the five studies compared their approach to models without self-supervised pre-training and reported slight relative improvements in performance of 1.12%^67^ and 0.690%^63^.

### Combined approaches

Eleven studies found creative ways to combine different self-supervised learning strategies to pre-train their medical imaging models (Table 1). Over half of these studies (6/11) combined contrastive with generative approaches. With the exception of Ke et al.’s work^68^, which uses a CycleGAN for histopathology slide stain normalization, all studies utilized an autoencoder as their generative model when combined with contrastive strategies. A combination of contrastive and innate relationships was used in three studies. The innate relationship tasks range from augmentation prediction and patch positioning prediction^69^, rotation prediction^70^, and ultrasound video to speech correspondence prediction^53^. For the remaining two studies, Cornelissen et al. used a conditional GAN, and trained the generator network on endoscopic images of the esophagus to recolorize, inpaint and generate super-resolution images^71^. Because their tasks consisted of both inpainting (self-prediction) and super-resolution (generative), their approach was considered combined. Haghighi et al. combined three different SSL strategies (generative, innate relationship, self-prediction) by first training an auto-encoder and group instances with similar appearances based on the latent representations from the auto-encoder^72^. Then, the authors randomly cropped image patches at a fixed coordinate for all instances in the same group and assigned a pseudo label to the cropped patches at each coordinate. Finally, the cropped patches were randomly perturbed and a restoration autoencoder was trained simultaneously with a pseudo label classification objective. Eight of the studies that combined different strategies compared self-supervised pre-training with purely supervised approaches, all of which reported performance improvement (0.140–8.29%).

## Discussion

This review aims to aggregate the collective knowledge of prior works that applied SSL to medical classification tasks. By synthesizing the relevant literature, we provide consistent definitions for SSL techniques, categorize prior work by their pre-training strategies, and provide implementation guidelines based on lessons learned from prior work. While five studies reported a slight decrease in performance (0.980–4.51%), the majority of self-supervised pre-trained models led to a relative increased performances of 0.216–32.6% AUROC, 0.440–29.2% accuracy, and 0.137–14.3% F1 score over the same model architecture without SSL pre-training, including both ImageNet and random initialization (Fig. 4e). In Fig. 4c we show a comparison of different SSL strategies on the same downstream task, which suggests that a combined strategy tends to outperform other self-supervised categories. However, it is important to note that combined strategies are typically the proposed method in the manuscripts, and thus publication bias might have resulted in this trend. In Fig. 4d we additionally plot the performance of the two main types of fine-tuning strategies on the same task, and the graph tends to indicate that end-to-end fine-tuning leads to better performance regardless of the dataset size. In the presence of relevant data, we recommend implementing self-supervised learning strategies for training medical image classification models since our literature review indicated that self-supervised pre-training generally results in better model performance, especially when annotations are limited (Table 1).

The types of medical images utilized for model development as well as the downstream classification task encompassed a wide range of medical domains and applications (Fig. 4a, b). The vast majority of the studies explored the clinical domain of radiology (47/79), of which 27 were focused on investigating abnormalities on chest imaging such as pneumonia, COVID-19, pleural effusion and pulmonary embolism (see Table 1). The choice of this domain is likely a combination of the availability of large-scale public chest datasets such as CheXpert^73^, RSPECT^74^, RadFusion^75^ and MIMIC-CXR^76^, as well as the motivation to solve acute or emerging healthcare threats, which was the case during the coronavirus pandemic^45,46,48,67,77–83^. The second most prevalent clinical domain was pathology (12/79). Similar to radiology, this field is centered around medical imaging in the form of whole slide images. The tasks were focused on histopathological classification, where the majority focused on colorectal cancer classification^68,84–87^. The remaining studies explored multiple other tasks and many focused on classification of malignant disorders such as breast cancer^56,61,88^, skin cancer^89^, and lung cancer^59^. A particularly interesting medical task that was explored was classification of psychiatric diseases or psychiatric traits using fMRI^57,62,90^. Current limited knowledge and understanding of possible imaging features arising in psychiatric diseases constitutes a major clinical challenge to making local annotations such as bounding boxes or segmentations on brain scans. In this case both Osin et al. and Hashimoto demonstrated that training a self-supervised framework could be beneficial to generate representative latent features of brain fMRIs before fine-tuning on image-level class labels^57,90^.

A majority of the included studies lacked strong baselines and ablation experiments. Even though 60 out of 79 studies compared their results with purely supervised baselines, only 33 studies reported comparisons with another self-supervised learning strategy. Of the 33 studies, 26 compared with a self-supervised category that differs from their best performing model. Among the SSL baselines, SimCLR was most frequently compared (16/26), followed by autoencoders (11/26) and MoCo (9/26). Furthermore, only 18 out of 79 studies indicated use of natural image pre-trained weights, either supervised or self-supervised, to initiate their model for subsequent in-domain self-supervised pre-training. Lastly, merely 13 studies compared performance between classification on extracted features to end-to-end fine-tuning, two of which did not report numerical results. Of the 11 studies that quantitatively reported performance, eight found end-to-end fine-tuning to outperform training a new classifier on extracted features (Fig. 4d). Since self-supervised learning for medical images is a promising yet nascent research area and the optimal strategies for training these models are still to be explored, researchers should systematically investigate different categories of self-supervised learning for their medical image datasets, in addition to fine-tuning strategy and pre-trained weights. Researchers should also test their newly developed strategies on multiple datasets, ideally on different modalities and medical imaging domains.

### Implementation guidelines

Definitive conclusions on the optimal strategy for medical images cannot be made since only a subset of studies made comparisons between different types of self-supervised learning strategies. Furthermore, the optimal strategy may be dependent on a number of factors including the specific medical imaging domain, the size and complexity of the dataset, and the type of classification task^91,92^. Due to this heterogeneity, we encourage researchers to compare multiple self-supervised learning strategies for developing medical image classification models, especially in limited data regimes. Although self-supervised pre-training can be computationally demanding, many models pre-trained in a self-supervised manner on large-scale natural image datasets are publicly available and should be utilized. Azizi et al. have shown that SSL pre-trained models using natural images tend to outperform purely supervised pre-trained models^93^ for medical image classification, and continuing self-supervised pre-training with in-domain medical images leads to the best results. More recently, Azizi et al. found that using generic and large-scale *supervised* pre-trained models, such as BigTransfer^94^, can also benefit subsequent domain-specific self-supervised pre-training, and ultimately improve model performance and robustness for different medical imaging modalities^95^. Truong et al. have demonstrated the effectiveness of combining representations from multiple self-supervised methods to improve performance for three different medical imaging modalities^96^.

It is worth noting that representations learned using certain SSL strategies can be relatively more linearly separable, while representations from other strategies can achieve better performance when more layers or the entire model are fine-tuned. For instance, for natural image datasets, MoCo outperforms MAE by training a linear model on extracted features (linear probing), while MAE achieves better performance than MoCo as the number of fine-tune layers increases^41^. Likewise, Cornelissen et al. demonstrated that using representations from earlier layers can improve downstream classification of neoplasia in Barrett’s Esophagus^71^. Factors such as the degree of domain shift between SSL pre-training data and downstream task data could also affect the linear separability of the representations. Based on our aggregated results, we found that end-to-end fine-tuning generally leads to better performance for medical images (Fig. 4c). However, due to the lack of ablation studies from current work, we cannot determine whether fine-tuning only later layers of the model could lead to better performance relative to end-to-end fine-tuning. Furthermore, even though self-supervised learning strategies generate label-efficient representations, the learning process typically requires a relatively large amount of unlabeled data. For instance, reducing the number of pre-training images from 250k to 50k typically leads to a more than 10.0% drop in accuracy for downstream tasks, while reducing from 1 M to 250k leads to a 2.00–4.00% decrease^92^. Curating large-scale medical image datasets from multiple institutions is often challenged by the difficulty of sharing patient data while preserving patient privacy. Nevertheless, using federated learning, Yan et al. have demonstrated the possibility of training self-supervised models with data from multiple healthcare centers without the need for explicitly sharing data, and have shown improvement in robustness and performance over models trained using data from only one institution^97^.

**Fig. 5:**
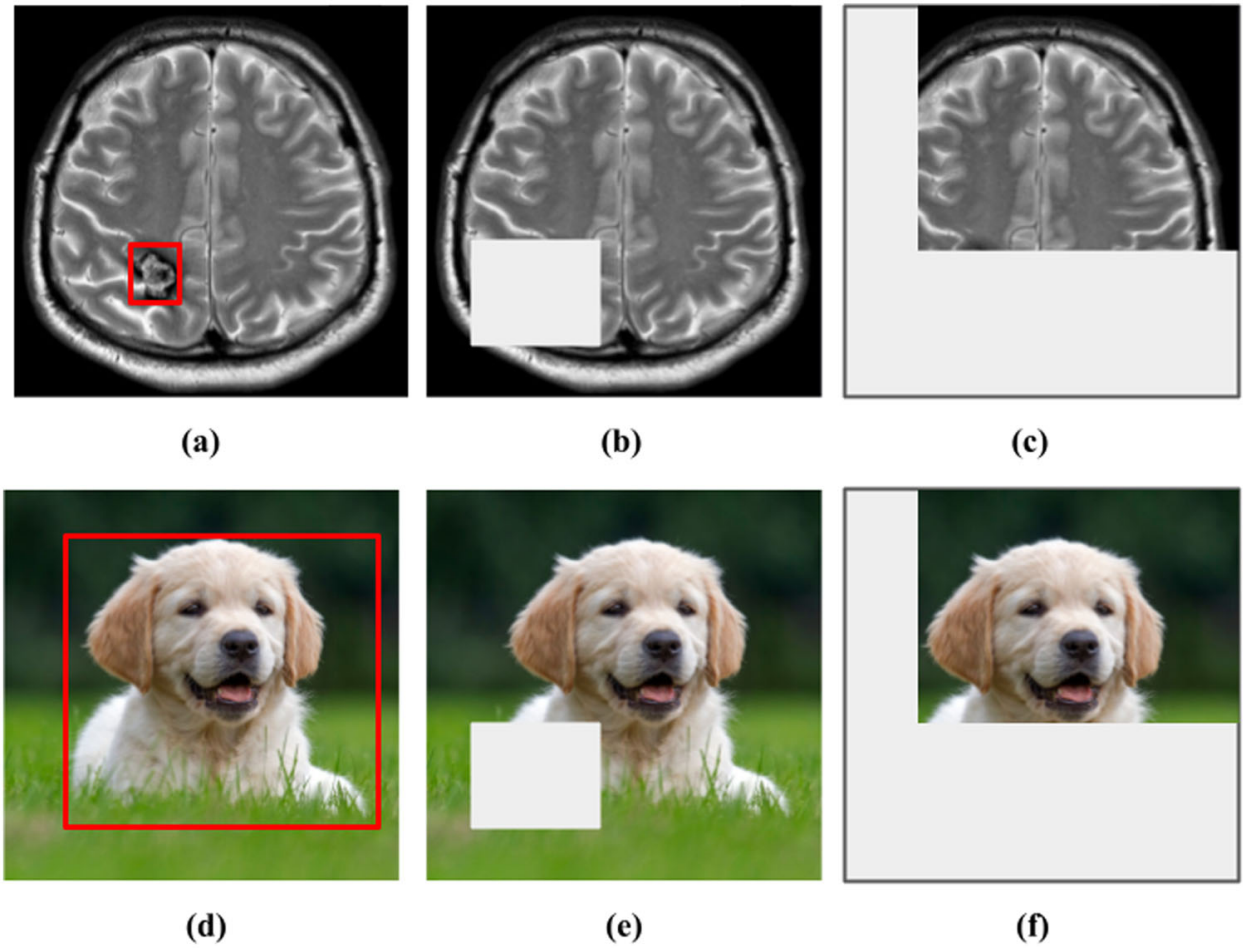
Examples of augmentations and transformations that alter the semantic meaning of medical images^144^ but not natural images^145^. **a** The image shows a T2-weighted brain MRI with a cavernoma in the right parietal lobe (bounded in red). **b** and **c** Masking and cropping operations can obscure the cavernoma and alter the semantic meaning of the image, as the MRI-scan no longer exhibits any abnormality. **d** Image of a dog (bounded in red), (**e**) and (**f**) Masking and cropping operations do not obscure the dog nor alter the semantic meaning of the image.

The field of self-supervised learning for computer vision is constantly and rapidly evolving. While many self-supervised methods have led to state-of-the-art results when fine-tuned on natural image datasets, how translatable these results are to medical datasets is unclear, mainly due to the unique properties of medical images. For instance, many contrastive-based strategies have been developed based on the assumption that the class-defining object is the main focus of an image, and thus variations caused by image transformations should not alter the image’s semantic meanings (Fig. 5). Therefore, these methods typically apply strong transformations to the original image and encourage the model to learn similar global representations for images with similar semantic meanings. However, the assumption made for natural images is not necessarily valid for medical images for two reasons. First, medical images have high inter-class visual similarities due to the standardized protocols for medical image acquisition and the homogeneous nature of human anatomy. Second, within the medical imaging domain, the semantic meaning of interest is rarely an object such as the anatomical organ, but is rather the presence or absence of pathological abnormalities within that organ or tissue. Many abnormalities are characterized by very subtle and localized visual cues, which can become ambiguous or obscured by augmentations (Fig. 5c). The random masking operation often utilized by self-prediction self-supervised learning methods may also alter a medical image’s semantic meaning by removing image regions with diseases or abnormalities (Fig. 5b). Recent work has demonstrated the benefit of using learned visual word masks^98,99^ or spatially constrained crops^100,101^ to encourage representational invariance with semantically more similar regions of an image. In a similar vein, we believe that augmentation strategies tailored for the nature of medical images during self-supervised learning is a research area that warrants further exploration.

The unique properties of medical images can be leveraged to design self-supervised learning methods more suitable for specific downstream tasks. For instance, instead of forming positive pairs with different augmented versions of the same image during contrastive learning, one can improve positive sampling according to the similarity between a patient’s clinical information. In fact, several studies have shown performance improvement when constructing positive pairs with slices from the same CT series^102^, images from the same imaging study^103^, images from the same patient^93^ and images from patients with similar age^62^. Future research should explore other strategies for defining positive pairs, such as leveraging patient demographics or medical history information. The unique attributes of medical images can also be utilized for creating relevant pre-text tasks. Rivail et al. proposed a self-supervised approach to model disease progression by estimating the time interval between pairs of optical coherence tomography (OCT) scans from the same patient^104^. Involving additional modalities during self-supervised learning has also been shown to improve a model’s performance when fine-tuned for downstream tasks. For example, Taleb et al. proposed a multimodal contrastive learning strategy between retinal fundus images and genetics data and showed improvement in performance over single modality pre-training^105^. Jiao et al. cleverly leveraged the correlation between fetal ultrasonography and the narrative speech of the sonographer to create a pre-text task for self-supervision, and subsequently used the learned representations for downstream standard plane classification on sonograms^53^. Furthermore, many medical imaging modalities have corresponding radiology reports that contain detailed descriptions of the medical conditions observed by radiologists. Several studies have utilized these medical reports to provide supervision signals during self-supervised learning and shown label efficiency for downstream tasks^60,106^. By leveraging radiology reports, Huang et al. demonstrated the model’s ability to localize chest abnormalities on chest x-rays without segmentation labels and revealed the possibility of zero-shot learning by converting the classification classes into textual captions and framing the image classification task as measuring the image-text similarity^107^. However, currently there are very few publicly available medical image datasets with corresponding radiology reports, largely due to the difficulties in preserving patient privacy. Therefore, these multi-modal self-supervised learning strategies are limited to implementation within a healthcare system until more datasets with medical image and report pairs are publicly released. Overall, the flexibility in creating self-supervised methods as well as the adaptability and transferability to multiple medical domains highlights the importance and utility of self-supervised techniques in clinical applications.

### Limitations

For this review paper, publication bias can be a considerable limitation due to disproportionately reported positive results in the literature, which can lead to overestimation of the benefits of self-supervised learning. We also limited our search to only consider papers published after 2012, which excluded papers that applied self-supervised learning prior to the era of deep learning for computer vision^108^. Furthermore, we are unable to aggregate or statistically compare the effects of each self-supervised learning strategy on performance gain, since the included studies use different imaging modalities, report different performance metrics, and investigate different objectives. In addition, subjectivity may have been introduced when categorizing the self-supervised learning strategy in each paper, especially for studies that implemented novel, unconventional, or a mixture of methods. Lastly, our study selection criteria only included literature for the task of medical image classification, which limits the scope of this review paper, since we recognize that self-supervised pre-trained models can also be fine-tuned for other important medical tasks, including segmentation, regression, and registration.

### Future research

Results from this systematic review have revealed that SSL for medical image classification is a growing and promising field of research across multiple medical disciplines and imaging modalities. We found that self-supervised pre-training generally improves performance for medical imaging classification tasks over purely supervised methods. We categorized the SSL approaches used in medical imaging tasks as a framework for methodologic communication and synthesized benefits and limitations from literature to provide recommendations for future research. Future studies should include direct comparisons of different self-supervised learning strategies using shared terminology and metrics whenever applicable to facilitate the discovery of additional insights and optimal approaches.

## Methods

This systematic review was conducted based on the PRISMA guidelines^109^.

### Search strategy

A systematic literature search was implemented in three literature databases: PubMed, Scopus and ArXiv. The key search terms were based on a combination of two major themes: “self-supervised learning” and “medical imaging”. Search terms for medical imaging were not limited to radiological imaging but were also broadly defined to include imaging from all medical fields, i.e., fundus photography, whole slide imaging, endoscopy, echocardiography, etc. Since we specifically wanted to review literature on the task of medical image classification, terms for other computer vision tasks such as segmentation, image reconstruction, denoising and object detection were used as exclusion criteria in the search. The search encompassed papers published between January 2012 and May 2021. The start date was considered appropriate due to the rising popularity of deep learning for computer vision since the 2012 ImageNet challenge. The complete search string for all three databases is provided in Supplementary Methods.

We included all research papers in English that used self-supervision techniques to develop deep learning models for medical image classification tasks. The research papers had to be original research in the form of journal articles, conference proceedings or extended abstracts. We excluded any publications that were not peer-reviewed. Applicable self-supervision techniques were defined according to the different types presented in the “terminology and strategies in self-supervised learning” section. We included only studies that applied the deep learning models to a downstream medical image classification task, i.e, it was not sufficient for the study to have developed a self-supervision model on medical images. In addition, the medical image classification task had to be a clinical task or clinically relevant task. For example, the downstream task of classifying the frame number in a temporal sequence of frames from echocardiography^110^ was not considered a clinically relevant task.

We excluded studies that used semi-supervised learning or any amount of manually curated labels during the self-supervision step. We also excluded studies that investigated only regression or segmentation in their downstream tasks. Furthermore, we excluded any studies where the self-supervised pre-trained model was not directly fine-tuned for classification after pre-training. Studies that used non-human medical imaging data (i.e., veterinarian medical images) were also excluded.

### Study selection

The Covidence software (www.covidence.org) was used for screening and study selection. After the removal of duplicates, studies were screened based on title and abstract, and then full texts were obtained and assessed for inclusion. Study selection was performed by three independent researchers (S.-C.H., A.P., and M.J.), and disagreements were resolved through discussion. In cases where consensus could not be achieved a fourth arbitrating researcher was consulted (A.S.C.).

### Data extraction

For benchmarking the existing approaches we extracted the following data from each of the selected articles: (a) self-supervised learning strategy, (b) year of publication, (c) first author, (d) imaging modality, (e) clinical domain, (f) outcome/task, (g) combined method, (h) self-supervised framework, (i) strategy for fine-tuning, (j) performance metrics, (k) SSL performance, (l) supervised performance, and (m) difference in SSL and supervised performance (Table 1). We also computed the relative difference in performance between the supervised and self-supervised model on the p) full dataset and q) subset. We classified the specific self-supervised learning strategy based on the definitions in the section “Terminology and strategies in self-supervised learning”. We extracted AUROC whenever this metric was reported, otherwise we prioritize accuracy over F1 score and sensitivity. When the article contained results from multiple models (i.e., ResNet and DenseNet), metrics from the experiment with the best average performing self-supervised model were extracted. When the authors presented results from several clinical tasks, we chose tasks that best corresponded to the title and objective of the manuscript. If the tasks were deemed equal, we picked the task where the chosen SSL model had the highest performance. We picked supervised baseline with the same model architecture and pre-training dataset for performance comparison. If the author did not report performance from a supervised model that used the same pre-training dataset, preference was given to the ImageNet pre-trained model over a randomly initialized one. The pre-training dataset used by the self-supervised and supervised model are recorded in the Supplementary Table 1. When papers report results on many percentages of fine-tuning (i.e., 1%, 10%, 100%), we pick the lowest and highest to study the label-efficiency of self-supervised learning methods. We also provide in Supplementary Table 1 additional technical details including model architecture, dataset details, number of training samples, comparison to selected baselines and performance on subsets of data. These items were extracted to enable researchers to find and compare current self-supervised studies in their medical field or input modalities of interest.

## Supplementary information

Supplemental Data

Supplemental Methods

## Data Availability

The authors declare that all data supporting the findings of this study are available within the paper and its Supplementary Information files.
