# Supplementary material for: Self-supervised learning for medical image classification: a systematic review and implementation guidelines
Source: NPJ Digit Med. 2023 Apr 26;6:74. doi: 10.1038/s41746-023-00811-0 (PMC10131505; doi:10.1038/s41746-023-00811-0)
Supplement: Supplementary file 2 — Supplemental Methods [file 41746_2023_811_MOESM2_ESM.pdf]

Supplementary Table

We provide in Supplementary Table 1: a) paper title, b) first author, c) year of publication, d) open-source code link, e) supervised baseline weight initialization strategy, f) if multiple modalities are used during self-supervised learning, g) model architecture, h) dataset used for self-supervised learning, i) dataset used for downstream tasks, j) number of samples in dataset used for self-supervised learning, k) number of samples in downstream task dataset, l) unit of measurement for samples, m) if the model was initialized with ImageNet, n) if multiple tasks or datasets were used, o) the authors compared end-to-end fine-tuning with classification on extracted features, p) the smallest % of subset the authors fine-tuned their pretrained model on, q) performance of SSL pre-trained model when fine-tuned with smallest subset of training data, r) performance of supervised pre-trained model when fine-tuned with smallest subset of training data, s) relative performance difference between supervised and SSL pretrained model when fine-tuned on smallest subset of training data, t) performance when using extracted features for downstream task, u) performance when using end-to-end fine-tuning, v) if additional SSL baselines are compared, and w-ah) the best reported SSL model from each category and their corresponding performances. If results from multiple models were reported, the architecture for the best performing self-supervised model were extracted. Similarly, if multiple datasets were used to evaluate downstream tasks performance, we pick the dataset where the chosen SSL model achieves the highest performance. We pick the best performance model from each SSL category based on the chosen task (Table 1f), and extract performance from the highest percentage of fine-tuning data. These items were extracted to enable researchers to find and compare current SSL studies in their medical field or input modalities of interest.

Supplementary Methods

Systematic search string on PubMed

((self-supervis*[Text Word] OR “self supervis*”[Text Word] OR “contrastive learning”[Text Word] OR contrastive-learning[Text Word] OR “contrastive loss”[Text Word] OR contrastive-loss[Text Word] OR “contrastive training”[Text Word] OR contrastive-training[Text Word] OR “noise contrastive estimation”[Text Word] OR “pretext learning”[Text Word] OR pretext-learning[Text Word] OR “pre-text learning”[Text Word] OR “pretext task”[Text Word] OR pretext-task[Text Word] OR “pre-text task”[Text Word]) AND (“medical imag*”[Text Word] OR “diagnostic imag*”[Text Word] OR "radiology imag*"[Text Word] OR "radiological imag*"[Text Word] OR "radiography"[MeSH Terms] OR "diagnostic imaging"[MeSH Terms] OR “CT scan*”[Text Word] OR CT-scan*[Text Word] OR “computed tomography”[Text Word] OR “computer-assisted tomography”[Text Word] OR “CAT scan*”[Text Word] OR CAT-scan*[Text Word] OR “PET scan*”[Text Word] OR “positron emission tomography”[Text Word] OR FDG-PET[Text Word] OR PET-CT[Text Word] OR PET/CT[Text Word] OR "positron-emission tomography"[MeSH Terms] OR "Positron Emission Tomography Computed Tomography"[MeSH Terms] OR "tomography, x-ray computed"[MeSH Terms] OR MRI[Text

Word] OR “magnetic resonance imaging”[Text Word] OR “MR scan*”[Text Word] OR MR-scan*[Text Word] OR “MRI scan*”[Text Word] OR MRI-scan*[Text Word] OR "Magnetic Resonance Imaging"[MeSH Terms] OR x-ray*[Text Word] OR radiograph*[Text Word] OR "Radiography"[MeSH Terms] OR "X-Rays"[MeSH Terms] OR mammogra*[Text Word] OR "mammography"[MeSH Terms] OR fluoroscopy[Text Word] OR "fluoroscopy"[MeSH Terms] OR Ultrasound[Text Word] OR ultrasonograph*[Text Word] OR "Ultrasonography"[MeSH Terms] OR sonograph*[Text Word] OR echocardio*[Text Word] OR "Echocardiography"[MeSH Terms] OR spect-ct[Text Word] OR “single-photon emission computed tomography”[Text Word] OR “spect scan” [Text Word] OR "Single Photon Emission Computed Tomography Computed Tomography"[MeSH Terms] OR endoscop*[Text Word] OR colonoscop*[Text Word] OR gastroscop*[Text Word] OR "Endoscopy"[MeSH Terms] OR "Colonoscopy"[MeSH Terms] OR "Gastroscopy"[MeSH Terms] OR Scintigraphy[Text Word] OR “gamma scan”[Text Word] OR “perfusion scan”[Text Word] OR "radionuclide imaging"[MeSH Terms] OR "Ventilation-Perfusion Scan"[MeSH Terms] OR “whole slide imaging”[Text Word] OR “microscope slide”[Text Word] OR “microscope slides”[Text Word] OR “pathology imaging”[Text Word] OR “pathology slid*”[Text Word] OR Microscope[Text Word] OR microscopy[Text Word] OR "Microscopy"[MeSH Terms] OR ophthalmoscope[Text Word] OR ophthalmoscopy[Text Word] OR “fundus photography”[Text Word] OR “fundus imaging”[Text Word] OR “retinal image”[Text Word] OR "Microscopy"[MeSH Terms] OR "Ophthalmoscopes"[MeSH Terms] OR dermatoscope[Text Word] OR dermatoscopy[Text Word] OR "dermoscopy"[MeSH Terms]) NOT (Registration[Text Word] OR Segmentation[Text Word] OR Reconstruction[Text Word] OR Denoising[Text Word] OR “Object detection”[Text Word] OR VQA[Text Word] OR “Visual question answering”[Text Word]) AND ("2012/01/01"[PDAT] : "2022/01/01"[PDAT])

Systematic search string on ArXiv

http://export.arxiv.org/api/query?search_query=%28%22noise+contrastive+estimation%22+OR+abs:%22noise+contrastive+estimation%22+OR+ti:self-supervise+OR+abs:self-supervise+OR+ti:self-supervised+OR+abs:self-supervised+OR+ti:self-supervision+OR+abs:self-supervision+OR+ti:%22self+supervise%22+OR+abs:%22self+supervise%22+OR+ti:%22self+supervised%22+OR+abs:%22self+supervised%22+OR+ti:%22self+supervision%22+OR+abs:%22self+supervision%22+OR+ti:%22contrastive+learning%22+OR+abs:%22contrastive+learning%22+OR+ti:contrastive-learning+OR+abs:contrastive-learning+OR+ti:%22contrastive+loss%22+OR+abs:%22contrastive+loss%22+OR+ti:contrastive-loss+OR+abs:contrastive-loss+OR+ti:%22contrastive+training%22+OR+abs:%22contrastive+training%22+OR+ti:contrastive-training+OR+abs:contrastive-training+OR+ti:%22pretext+task%22+OR+abs:%22pretext+task%22+OR+ti:pretrext-task+OR+abs:pretext-task+OR+ti:%22pre-text+task%22+OR+abs:%22pre-text+task%22+OR+ti:%22pretext+learning%22+OR+abs:%22pretext+learning%22+OR+ti:pretrext-learning+OR+abs:pretext-learning+OR+ti:%22pre-text+learning%22+OR+abs:%22pre-text+learning%22%29+AND+%28ti:%22medical+image%22+OR+abs:%22medical+image%22

+OR+ti:%22medical+images%22+OR+abs:%22medical+images%22+OR+ti:%22medical+imaging%22+OR+abs:%22medical+imaging%22+OR+ti:%22diagnostic+image%22+OR+abs:%22diagnostic+image%22+OR+ti:%22diagnostic+images%22+OR+abs:%22diagnostic+images%22+OR+ti:%22diagnostic+imaging%22+OR+abs:%22diagnostic+imaging%22+OR+ti:%22radiology+image%22+OR+abs:%22radiology+image%22+OR+ti:%22radiology+images%22+OR+abs:%22radiological+imaging%22+OR+ti:%22radiological+imaging%22+OR+abs:%22radiology+images%22+OR+ti:%22radiology+imaging%22+OR+abs:%22radiology+imaging%22+OR+ti:%22ct+scan%22+OR+abs:%22ct+scan%22+OR+ti:%22ct+scans%22+OR+abs:%22ct+scans%22+OR+ti:ct-scan+OR+abs:ct-scan+OR+ti:ct-scans+OR+abs:ct-scans+OR+ti:%22computed+tomography%22+OR+abs:%22computed+tomography%22+OR+ti:%22computer-assisted+tomography%22+OR+abs:%22computer-assisted+tomography%22+OR+ti:%22cat+scan%22+OR+abs:%22cat+scan%22+OR+ti:%22cat+scans%22+OR+abs:%22cat+scans%22+OR+ti:cat-scan+OR+abs:cat-scan+OR+ti:cat-scans+OR+abs:cat-scans+OR+ti:%22pet+scan%22+OR+abs:%22pet+scan%22+OR+ti:%22pet+scans%22+OR+abs:%22pet+scans%22+OR+ti:%22positron+emission+tomography%22+OR+abs:%22positron+emission+tomography%22+OR+ti:fdg-pet+OR+abs:fdg-pet+OR+ti:pet-ct+OR+abs:pet-ct+OR+ti:pet/ct+OR+abs:pet/ct+OR+ti:spect-ct+OR+abs:spect-ct+OR+ti:%22single-photon+emission+computed+tomography%22+OR+abs:%22single-photon+emission+computed+tomography%22+OR+ti:mri+OR+abs:mri+OR+ti:%22magnetic+resonance+imaging%22+OR+abs:%22magnetic+resonance+imaging%22+OR+ti:%22mr+scan%22+OR+abs:%22mr+scan%22+OR+ti:%22mr+scans%22+OR+abs:%22mr+scans%22+OR+ti:mr-scan+OR+abs:mr-scan+OR+ti:mr-scans+OR+abs:mr-scans+OR+ti:mri-scan+OR+abs:mri-scan+OR+ti:mri-scans+OR+abs:mri-scans+OR+ti:x-ray+OR+abs:x-ray+OR+ti:x-rays+OR+abs:x-rays+OR+ti:radiograph+OR+abs:radiograph+OR+ti:radiographs+OR+abs:radiographs+OR+ti:mammogram+OR+abs:mammogram+OR+ti:mammograms+OR+abs:mammograms+OR+ti:mammography+OR+abs:mammography+OR+ti:fluoroscopy+OR+abs:fluoroscopy+OR+ti:ultrasound+OR+abs:ultrasound+OR+ti:ultrasonogram+OR+abs:ultrasonogram+OR+ti:ultrasonograms+OR+abs:ultrasonograms+OR+ti:ultrasonography+OR+abs:ultrasonography+OR+ti:sonogram+OR+abs:sonogram+OR+ti:sonograms+OR+abs:sonograms+OR+ti:sonography+OR+abs:sonography+OR+ti:echocardiogram+OR+abs:echocardiogram+OR+ti:echocardiograms+OR+abs:echocardiograms+OR+ti:echocardiography+OR+abs:echocardiography+OR+ti:endoscope+OR+abs:endoscope+OR+ti:endoscopic+OR+abs:endoscopic+OR+ti:endoscopy+OR+abs:endoscopy+OR+ti:endoscopies+OR+abs:endoscopies+OR+ti:colonoscope+OR+abs:colonoscope+OR+ti:colonoscopy+OR+abs:colonoscopy+OR+ti:colonoscopies+OR+abs:colonoscopies+OR+ti:gastroscopy+OR+abs:gastroscopy+OR+ti:gastroscope+OR+abs:gastroscope+OR+ti:gastroscopies+OR+abs:gastroscopies+OR+ti:scintigraphy+OR+abs:scintigraphy+OR+ti:%22gamma+scan%22+OR+abs:%22gamma+scan%22+OR+ti:%22gamma+scans%22+OR+abs:%22gamma+scans%22+OR+ti:%22perfusion+scan%22+OR+abs:%22perfusion+scan%22+OR+ti:%22perfusion+scans%22+OR+abs

:%22perfusion+scans%22+OR+ti:%22whole+slide+imaging%22+OR+abs:%22whole+slide+imaging%22+OR+ti:%22microscope+slide%22+OR+abs:%22microscope+slide%22+OR+ti:%22microscope+slides%22+OR+abs:%22microscope+slides%22+OR+ti:%22pathology+imaging%22+OR+abs:%22pathology+imaging%22+OR+ti:%22pathology+slide%22+OR+abs:%22pathology+slide%22+OR+ti:%22pathology+slides%22+OR+abs:%22pathology+slides%22+OR+ti:microscope+OR+abs:microscope+OR+ti:microscopy+OR+abs:microscopy+OR+ti:ophthalmoscope+OR+abs:ophthalmoscope+OR+ti:ophthalmoscopy+OR+abs:ophthalmoscopy+OR+ti:%22fundus+photography%22+OR+abs:%22fundus+photography%22+OR+ti:%22fundus+imaging%22+OR+abs:%22fundus+imaging%22+OR+ti:%22retinal+image%22+OR+abs:%22retinal+image%22+OR+ti:dermatoscope+OR+abs:dermatoscope+OR+ti:dermatoscopy+OR+abs:dermatoscopy%29+ANDNOT+%28abs:reconstruction+OR+ti:reconstruction+OR+abs:denoising+OR+ti:denoising+OR+abs:segmentation+OR+ti:segmentation+OR+abs:%22object+detection%22+OR+ti:%22object+detection%22+OR+abs:registration+OR+ti:registration+OR+abs:vqa+OR+ti:vqa+OR+abs:%22visual+question+answering%22+OR+ti:%22visual+question+answering%22%29&date-year=&date-filter_by=date_range&date-from_date=2012-01-01&date-to_date=2022-01-01&date-date_type=submitted_date&max_results=500

Systematic search string on Scopus

((TITLE-ABS-KEY ("self-supervis*" OR "self supervis*") OR TITLE-ABS-KEY ("contrastive learning" OR contrastive-learning) OR TITLE-ABS-KEY ("contrastive loss" OR contrastive-loss) OR TITLE-ABS-KEY ("contrastive training" OR contrastive-training) OR TITLE-ABS-KEY ("noise contrastive estimation") OR TITLE-ABS-KEY ("pretext learning" OR pretext-learning OR "pre-text learning") OR TITLE-ABS-KEY ("pretext task" OR pretext-task OR "pre-text task"))) AND ((TITLE-ABS-KEY ("medical imag*" OR "diagnostic imag*" OR "radiolog* imag*") OR TITLE-ABS-KEY ("CT scan*" OR ct-scan* OR "computed tomography" OR "computer-assisted tomography" OR "CAT scan*" OR cat-scan*) OR TITLE-ABS-KEY ("PET scan*" OR "positron emission tomography" OR fdg-pet OR pet-ct OR "PET/CT") OR TITLE-ABS-KEY (mri OR "magnetic resonance imaging" OR "MR scan*" OR mr-scan* OR "MRI scan*" OR mri-scan*) OR TITLE-ABS-KEY (x-ray* OR radiograph*) OR TITLE-ABS-KEY (mammogram OR mammography) OR TITLE-ABS-KEY (fluoroscopy) OR TITLE-ABS-KEY (ultrasound OR ultrasonograph* OR sonograph* OR echocardio*) OR TITLE-ABS-KEY (spect-ct OR "SPECT Scan" OR "single-photon emission computed tomography") OR TITLE-ABS-KEY (endoscop* OR colonoscop* OR gastroscop*) OR TITLE-ABS-KEY (scintigraphy OR "gamma scan" OR "perfusion scan") OR TITLE-ABS-KEY ("whole slide imaging" OR "microscope slid*" OR "pathology imaging" OR "pathology slid*") OR TITLE-ABS-KEY (microscope OR microscopy) OR TITLE-ABS-KEY (ophthalmoscope OR ophthalmoscopy OR "fundus photography" OR "fundus imaging" OR "retinal image") OR TITLE-ABS-KEY (dermatoscop*))) AND NOT ((TITLE-ABS-KEY (reconstruction) OR TITLE-ABS-KEY (denoising) OR TITLE-ABS-KEY (segmentation) OR TITLE-ABS-KEY ("object detection") OR TITLE-ABS-KEY (registration) OR TITLE-ABS-KEY (vqa) OR TITLE-ABS-KEY ("Visual Question Answering"))) AND (LIMIT-TO (PUBYEAR , 2022) OR LIMIT-TO (PUBYEAR , 2021) OR LIMIT-TO (PUBYEAR , 2020) OR LIMIT-TO (PUBYEAR ,

2019) OR LIMIT-TO (PUBYEAR , 2018) OR LIMIT-TO (PUBYEAR , 2017) OR LIMIT-TO (PUBYEAR , 2016) OR LIMIT-TO (PUBYEAR , 2015) OR LIMIT-TO (PUBYEAR , 2014) OR LIMIT-TO (PUBYEAR , 2013) OR LIMIT-TO (PUBYEAR , 2012))
